# Supplementary material for: Spatio-spectral metrics in electron energy loss spectroscopy as a tool to resolve nearly degenerate plasmon modes in dimer plasmonic antennas
Source: Nanophotonics. 2023 Jun 19;12(15):3089–98. doi: 10.1515/nanoph-2023-0153 (PMC11501496; doi:10.1515/nanoph-2023-0153)
Supplement: Supplementary file 1 — Supplementary Material Details [file j_nanoph-2023-0153_suppl_001.pdf]

Supplementary Material

Michal Horák*, Andrea Konečná, Tomáš Šikola, and Vlastimil Křápek*

Spatio-spectral metrics in electron energy loss spectroscopy as a tool to resolve nearly degenerate plasmon modes in dimer plasmonic antennas

S1 Localized surface plasmon resonances of individual disc

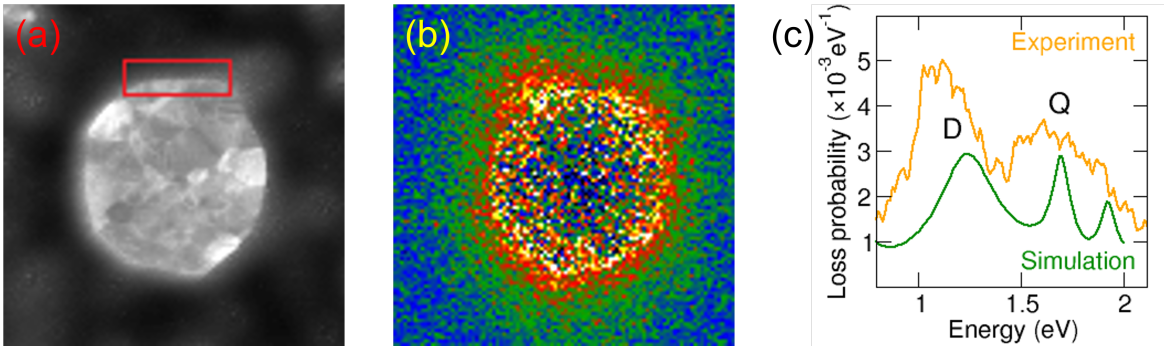

Fig. S1: (a) ADF image of the individual gold disc. The red rectangle denotes the region of interest in which the experimental EEL spectrum shown in panel c was recorded. (b) A map of the loss probability as obtained from the experiment at the energy of the dipole LSP (1.14 eV). (c) EEL spectra of the individual gold disc obtained from the experiment (orange) and simulations (green).

The individual gold disc was fabricated and characterized using the same methodology as utilized for the double discs. Fig. S1(a) shows the annular-dark-field (ADF) image of the disc. An experimental electron energy loss (EEL) map taken at the energy of the dipole localized surface plasmon resonance (LSPR) is shown in Fig. S1(b) and exhibits a circular symmetry. The experimental and calculated EEL spectra are shown in Fig. S1(c). The experimental spectrum was averaged over the area of interest denoted by the red rectangle in Fig. S1(a) and the calculated spectrum was taken for the distance of 30 nm from the disc. Both spectra correspond very well with each other and exhibit two distinct peaks attributed to the dipole (D) and quadrupole (Q) LSPR. The energy of the dipole LSPR as determined from the experiment (theory) reads 1.14 ± 0.10 eV (1.25 eV), respectively, and the energy of the quadrupole mode reads 1.62 ± 0.10 eV (1.69 eV). Importantly, the energy separation between D and Q modes is rather large and makes us confident that

***Corresponding author: Michal Horák, Andrea Konečná, Tomáš Šikola, Vlastimil Křápek,** Central European Institute of Technology, Brno University of Technology, Purkyňova 123, 612 00 Brno, Czech Republic, e-mail: michal.horak2@ceitec.vutbr.cz, krapek@vutbr.cz
Andrea Konečná, Tomáš Šikola, Vlastimil Křápek, Institute of Physical Engineering, Brno University of Technology, Technická 2, 616 69 Brno, Czech Republic

the LSP modes in the energy interval 0.8 – 1.5 eV analyzed with spatio-spectral metrics are significantly contributed by hybridized dipole modes with little to no contribution of the quadrupole modes.

S2 Non-negative matrix factorization

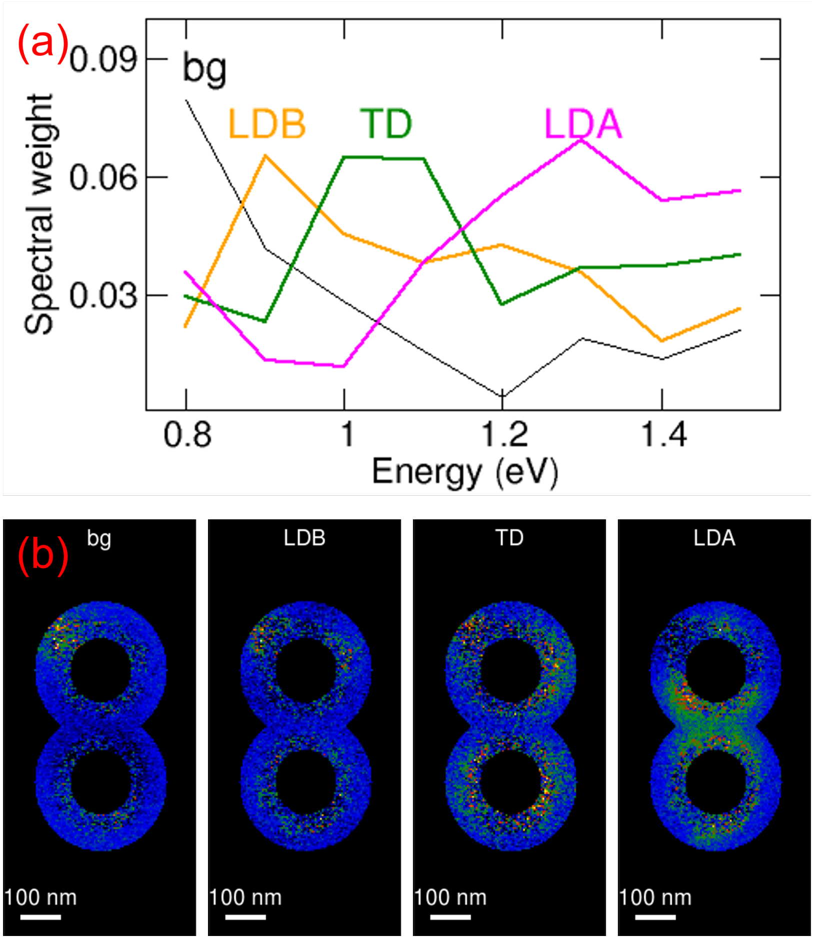

Fig. S2: (a) Spectral profiles of the weight of loss probability modes obtained from NNMF. (b) Spatial maps of the individual modes.

We examined the possibility to resolve and identify the nearly degenerate dipole LSPR supported by the disc-shaped dimer using non-negative matrix factorization (NNMF), a traditional technique of statistical analysis. We utilized the implementation of NNMF provided in Wolfram Mathematica software. When applied to the full field of view of the experimental EELS data cube, NNMF failed to provide a meaningful outcome due to large experimental noise. For this reason, we spatially restricted the analyzed data to two rings concentric with the plasmonic discs with a width of 100 nm, just as in the case of spatio-spectral metrics. We then applied NNMF with the number of modes set to 3, 4, and 5. The best results were obtained for 4 modes. Fig. S2 shows the spectral dependence of the mode weights and their spatial maps. Mode 1 corresponds to the background (bg) and modes 2, 3, 4 correspond to LDB, TD, and LDA LSPR, respectively. Their central energies are determined as 0.9 eV, 1.05 eV, and 1.3 eV, respectively. They agree reasonably well with the corresponding energies determined from the spatio-spectral metrics (see Table 1 in

the main text). However, the spatial maps of the modes provide only a little clue to their assignment to specific LSP modes. Instead, we rely on the assignment based on the spatio-spectral metrics described in the main text. Therefore, NNMF allows to resolve the LSP modes but in contrast to the metrics it does not allow to identify them. Finally, we remark that the identification of the LSP modes shall not be based only on the energy ordering, which can be altered due to effects such as slight ellipticity of the discs.
